# The Effect of Antioxidants on the Properties of Red Blood Cells From Patients With Sickle Cell Anemia

**DOI:** 10.3389/fphys.2019.00976

**Published:** 2019-08-13

**Authors:** Halima Al Balushi, Anke Hannemann, David Rees, John Brewin, John Stanley Gibson

**Affiliations:** ^1^Department of Veterinary Medicine, University of Cambridge, Cambridge, United Kingdom; ^2^Department of Paediatric Haematology, King’s College Hospital, King’s College London, London, United Kingdom

**Keywords:** antioxidant, sickle cell, dithiothreitol, *N*-acetylcysteine, quercetin, K^+^ permeability, sickling, phosphatidylserine exposure

## Abstract

Oxidative damage to red blood cells (RBCs) may contribute to pathogenesis of sickle cell anemia. Reducing the deleterious effects of oxidants by exposing RBCs to a number of antioxidants has been shown to have protective effects against lipid and protein peroxidation. We hypothesize that antioxidants may also have beneficial effects on the abnormal membrane permeability of sickle cells. Increased cation permeability of these cells encourages HbS polymerization by causing RBC dehydration and also leads to externalization of the prothrombotic aminophospholipid phosphatidylserine (PS). Three antioxidants with different mechanisms of action were investigated – dithiothreitol, *N*-acetylcysteine, and quercetin. All three were found to inhibit the main cation pathways responsible for dehydration – the deoxygenation-induced cation conductance (or P_sickle_), the Ca^2+^-activated K^+^ channel (or Gardos channel), and the K^+^-Cl^−^ cotransporter. They also reduced Ca^2+^-induced PS exposure and hemolysis. Findings provide evidence for additional beneficial actions of antioxidants in maintenance of rheology and reducing vascular adhesion and further inform the rationale for their clinical use.

## Introduction

Antioxidants are used in the treatment of many disorders notably neoplasia but also including sickle cell anemia (SCA), in which a phase 3 clinical trial of L-glutamine has recently been carried out (Niihara et al., 2018). Any effect on red blood cell (RBC) membrane permeability, however, has been largely ignored and is the subject of this report.

RBCs from patients with sickle cell anemia (of HbSS genotype; SCA) contain the abnormal hemoglobin (Hb) HbS. Compared to normal Hb, HbA, HbS results from a single base substitution in the seventh codon of the β globin gene, causing the replacement of glutamic acid with valine (Bunn and Forget, 1986). Loss of a negative charge at this site enables neighboring molecules of HbS to adhere following deoxygenation. The resultant long, rigid polymers of HbS initiate a concatenation of events ultimately resulting in the clinical signs observed in SCA patients. These fall largely into two groups: a chronic hemolytic anemia and acute episodes of vaso-occlusive ischemia. Complications include pain, acute chest syndrome, stroke, nephropathy, osteonecrosis, leg ulcers, and reduced lifespan, though both the frequency and severity of these problems vary markedly between patients (Steinberg, 1999; Rees et al., 2010).

SCA affects millions of people worldwide, especially in sub-Saharan Africa and India (Piel et al., 2013), where it results in considerable morbidity and mortality, as well as economic impact. Blood transfusion, antibiotic therapy, and pneumococcal vaccination all ameliorate complications; however, these apart, treatment remains largely supportive. While hydroxyurea has emerged as a specific licensed therapy (Platt et al., 1984; Charache et al., 1987), it is not without problems, and new effective therapies are keenly sought (Rees, 2011; Gibson et al., 2015).

An important feature of SCA is oxidative stress (Hebbel et al., 1982; Rice-Evans et al., 1986). Increased levels of reactive oxygen species are released from activated endothelial and white cells, from ischemia-reperfusion injury and from within RBCs, as HbS is more unstable than HbA breaking down into hemichromes and free iron (Hebbel et al., 1982, 1988; Rice-Evans et al., 1986; Aslan et al., 2000; Banerjee and Kuypers, 2004). It is also likely that antioxidant provision is reduced, both within RBCs and without circulating in plasma (Silva et al., 2013). For example, sickle red cells have a lower redox ratio with reduced levels of reduced nicotinamide adenine dinucleotide (Zerez et al., 1988; Al-Ali, 2002). Oxidant stress can result in lipid and protein damage with important pathogenic sequelae. It may also increase the solute permeability of RBCs, which is in itself problematical. It is not surprising, therefore, that the possible beneficial roles of antioxidant provision have received considerable attention.

Three abnormalities of HbS-containing RBCs are particularly relevant in the context of vascular adhesion and deformability. First, HbS polymerization deleteriously affects RBC shape and rheology (Hebbel et al., 1990), making it harder for RBCs to traverse the microvasculature. Second, HbS-containing RBCs expose higher levels of phosphatidylserine on their outer bilayer (Kuypers, 1998). This phospholipid is both prothrombotic and also increases adhesion to activated endothelial cells. Third, the unusually high cation permeability of HbS-containing RBCs mediates solute loss and dehydration, thus increasing intracellular HbS concentration (Lew and Bookchin, 2005). As the lag time to HbS polymerization is inversely proportional to a very high power of [HbS] (Eaton and Hofrichter, 1987), RBCs are therefore much more likely to sickle in hypoxic areas of the circulation. Three transport systems occupy a predominant role in mediating dehydration: the KCl cotransporter (KCC), the deoxygenation-induced cation conductance (sometimes called P_sickle_), and the Ca^2+^-activated K^+^ channel (or Gardos channel; Lew and Bookchin, 2005). KCC mediates coupled K^+^ and Cl^−^ loss, has elevated activity, and is abnormally regulated in sickle cells. P_sickle_ mediates Ca^2+^ entry and Mg^2+^ loss, which has three important effects: Ca^2+^ entry may activate the Gardos channel and also stimulate PS scrambling, while Mg^2+^ loss may increase KCC activity (Ortiz et al., 1990; Etzion et al., 1993).

Provision of antioxidant therapy to ameliorate the complications of SCA has been extensively investigated (Silva et al., 2013). Antioxidants have been shown to have a number of beneficial effects, protecting against RBC lipid peroxidation and increasing levels of reduced glutathione (GSH) while reducing levels of reactive oxygen species. Effects on RBC membrane permeability, however, have been largely unexplored. We postulate that antioxidant treatment may have important alternative beneficial effects on RBC function by reducing the permeability of the transport pathways mediating dehydration and by reducing PS exposure.

RBCs from SCA patients were exposed *in vitro* to three commonly used antioxidants – dithiothreitol (DTT), *N*-acetylcysteine (NAC), and quercetin (Silva et al., 2013) – and their effects on sickling, activities of P_sickle_, the Gardos channel and KCC, and PS exposure were investigated. Results reveal beneficial effects on HbS polymerization, inhibition of all three K^+^ dehydration pathways, and a reduction in externalized PS. Findings represent an additional rational reason to employ these reagents *in vivo* to ameliorate the complications of SCA.

## Materials and Methods

### Reagents

Clotrimazole was purchased from Calbiochem (Nottingham, Notts, UK). ^86^Rb^+^ was supplied by Perkin Elmer (Beaconsfield, Bucks, UK) and nitrogen by BOC Ltd. (Guildford, Surrey, UK). Fluorescein isothiocyanate-conjugated bovine lactadherin (LA-FITC) came from Innovative Research (Novi, MI, USA), supplied by Tebu-Bio Ltd. (Peterborough, UK). Other reagents were purchased from Sigma Chemical Co. (Poole, Dorset, UK).

### Sample Collection and Handling

Blood samples were taken at King’s College Hospital, London, with informed written consent from patients (and from their guardians if under 16) with sickle cell anemia (HbSS genotype) using the anticoagulant EDTA. Samples were anonymized blood samples, transported to Cambridge for laboratory assays, and used within 2 days. About half of the patients were being treated with hydroxyurea – there were no differences in the behavior of the cells comparing patients administered with this drug or not. Patients had not been transfused for at least 6 months, and none had a sickle cell crisis during this period. All research was conducted with ethical approval (NHS REC 16/LO/1309) and in accordance with the Helsinki Declaration of 1975, as revised in 2008.

### Solutions and Tonometry

The standard saline (Cl-MBS) comprised (in mM): NaCl 145, CaCl_2_ 1.1, glucose 5, and 3-(*N*-morpholino)-propane sulfonic acid (MOPS) 10 (pH 7.4 at 37°C; 290 ± 5 mOsm.kg^−1^ H_2_O). For experiments in which Cl^−^ dependence of K^+^ influx was examined, NO_3_^−^-containing salts replaced those containing Cl^−^ (N-MBS). For measurement of Gardos channel activity in Ca^2+^-loaded red cells (using the ionophore bromo-A23187, 6 μM), high potassium (HK)-containing MBS (HK-MBS) was used comprising (in mM) NaCl 70, KCl 80, and CaCl_2_ 0.01, together with MOPS (10 mM) and glucose (5 mM). In these experiments, the HK medium was used to prevent cell shrinkage, which would otherwise occur rapidly should channel activity be markedly stimulated. In flux experiments, to remove unincorporated radioisotope (^86^Rb^+^), the wash solution comprised isotonic MgCl_2_ (107 mM) and buffered with MOPS (10 mM), pH 7.4 at 4°C (Mg-MBS). Stock solutions of bumetanide (10 mM), ouabain (10 mM), and clotrimazole (5 mM) were prepared in 100 mM Tris base and distilled water and dimethylsulfoxide (DMSO), respectively. Whole blood was washed five times in N-MBS to remove Cl^−^, plasma, and buffy coat. For most experiments, red cells (20% hematocrit, Hct) were then pre-incubated in air at 37°C for 30 min in the absence or presence of antioxidants (which remained present throughout subsequent experimental manipulations). Red cell suspensions (still 20% Hct) in N-MBS were then placed in tonometers (Eschweiler, Kiel, Germany) and flushed with warm, humidified gas mixtures for 20 min at 37°C to equilibrate them at the requisite O_2_ tension before measurement of K^+^ influx and red cell morphology (Speake et al., 1997). Gas mixtures were made using a Wösthoff gas mixing pump (Speake et al., 1997). Three O_2_ tensions were used: 100 mmHg oxygen, 0 mmHg to oxygenate and deoxygenate red cells fully, and an intermediate tension of 30 mmHg at which HbS is about half saturated with oxygen, when intracellular oxidant production is highest (Abugo and Rifkind, 1994; Balagopalakrishna et al., 1996). For influx measurements, red cell suspensions were then diluted 10-fold into test tubes and still equilibrated at the same requisite O_2_ tension. To analyze red cell shape, RBC aliquots were fixed in saline containing 0.25% glutaraldehyde before examination under light microscopy counting typically around 300 cells (Hannemann et al., 2015).

### K^+^ Flux Measurements

To determine the activity of the K^+^ transport pathways, K^+^ influx was measured using ^86^Rb^+^ as a congener for K^+^ (Dunham and Ellory, 1981; Hannemann et al., 2011) at 37°C. Red cells were taken from tonometers and diluted 10-fold into saline, pre-equilibrated at the appropriate O_2_ tension at 260 mOsm.kg^−1^ (with the addition of 10% water to the appropriate standard MBSs) and pH 7 (conditions used to stimulate KCC activity). ^86^Rb^+^ was added in 150 mM KNO_3_ to give a final extracellular [K^+^] of 7.5 mM (except for experiments using bromo-A23187 – see below). Three flux conditions were used in the flux tubes: (1) Cl-MBS; (2) Cl-MBS with clotrimazole (CLT; 5 μM); and (3) N-MBS with CLT (5 μM). Ouabain (100 μM) and bumetanide (10 μM) were present in all experiments to obviate any K^+^ transport through the Na^+^/K^+^ pump and the Na^+^-K^+^-2Cl^−^ cotransporter, respectively. After incubation with radioisotope for 10 min, red cells were washed five times in ice-cold Mg-MBS wash solution to remove extracellular ^86^Rb^+^. Following the final wash, the cell pellet was lysed with Triton X-100 (0.1%), and protein precipitated with trichloroacetic acid (5%). Activity was then measured as Čerenkov radiation by liquid scintillation (Packard Tri-carb 2800TR, Perkin Elmer). P_sickle_ was assayed as the deoxygenation-induced, CLT-independent K^+^ influx measured in the absence of Cl^−^ (condition 3); Gardos channel activity as the CLT-sensitive (5 μM) K^+^ influx (using conditions 1 and 2); and KCC activity was assayed as Cl^−^-dependent K^+^ influx (using flux conditions 2 and 3). As CLT and A23187 were dissolved in DMSO, controls were all treated with the same concentration of this solvent (<0.5% final). Either microhematocrit determination or the cyanohemoglobin method was used to measure the hematocrit (Hct) with appropriate samples taken at the start of each experiment (Speake et al., 1997; Hannemann et al., 2011). For uptake experiments using bromo-A23187 to stimulate the Gardos channel through pharmacological Ca^2+^ loading of RBCs, the extracellular [Ca^2+^] was 10 μM. Following incubation with ^86^Rb^+^, RBC aliquots were placed in ice-cold wash solution layered over dibutylphthalate oil. RBCs were then spun through the oil by centrifugation at 15,000 *g* for 10 s, supernatant removed, the tubes washed with Mg-MBS, the oil removed, and tubes cleaned with cotton buds. The RBC pellet was then lysed with Triton X-100 (0.1%) as described for influx experiments.

### Non-electrolyte Hemolysis Assay

Previous work has shown that hemolysis of sickle cells in deoxygenated isosmotic non-electrolyte solutions provides a simple measure of P_sickle_ activity and RBC fragility (Browning et al., 2007; Milligan et al., 2013). The effect of antioxidants was therefore tested in this assay. Washed red cells were pre-incubated with antioxidants in Ca^2+^ free N-MBS (30 min, 37°C), pelleted, and resuspended in isosmotic sucrose solution (290 mOsm.kg^−1^, pH 7.0 at 37°C), whose composition followed that of the standard MBS but in which all salts were replaced with sucrose (255 mM), after which RBC suspensions were placed in Eschweiler tonometers and flushed with N_2_ for 60 min. To measure hemolysis, aliquots of the suspension were taken every 10 min, and intact red cells pelleted by centrifugation and the optical density (OD) of the supernatant measured at 540 nm. Values for 100% hemolysis were obtained from similar aliquots diluted into 0.1% Triton X-100.

### Measurement of Externalized Phosphatidylserine

To investigate the effects of antioxidants on (1) deoxygenated PS exposure or (2) PS exposure in Ca^2+^ loaded RBCs, RBC suspensions (0.5% HCT) in HK-HBS (in mM: NaCl 54, KCl 90, MgCl_2_ 0.15, inosine 10, and HEPES 10; pH 7.4 at 37°C; 290 ± 5 mOsm.kg^−1^) were first pre-incubated for 30 min with DTT (0.25 mM), NAC (10 mM), or quercetin (100 μM). For (1), they were then incubated in HK-HBS containing 1.1 mM CaCl_2_ and 1 mM vanadate in tonometers, deoxygenated by flushing them with humidified N_2_ up to 80 min in the continued presence or absence of antioxidants. Samples were taken at indicated times and PS labeled using LA-FITC. For (2), RBCs were then incubated HK-HBS (0.5% Hct, 30 min, 37°C) containing 2 mM EGTA and concentrations of total [Ca^2+^] of 1.35, 1.85, and 1.91 mM to clamp free extracellular [Ca^2+^] at 0.1, 0.6, and 1 μM, respectively, and permeabilized to Ca^2+^ with the Ca^2+^ ionophore bromo-A23187 (6 μM) in the absence or presence of antioxidants. The activity of bromo-A23187 was abrogated by adding 0.4 mM Co^2+^, and RBCs were pelleted and resuspended in HK-HBS containing 1 mM vanadate (pH 7.4 at RT). Exposed PS was labeled using LA-FITC (16 nM, 0.01% Hct) in HK-HBS containing 1 mM vanadate for 10 min at room temperature in the dark. RBCs were washed once and kept on ice until flow cytometry. LA-FITC was detected in the FL1 channel of a BD Accuri C6 flow cytometer using logarithmic gain. The positive fluorescent gate was set using red cells unlabeled with LA-FITC. For each measurement, 10,000 events were gated. PS positive cells were defined as all events fall within the preset FSC, SSC, and positive fluorescent gates.

### Statistics

Results are presented as means ± SEM of *n* observations in red cell samples taken from different individuals. Where appropriate, comparisons were made using unpaired and paired two-tailed Student’s *t* tests. Pearson correlation coefficients were calculated with GraphPad Prism (La Jolla, CA, USA). The level of significance used was *p* < 0.05. “Controls” refer to cells from SCA patients not exposed to antioxidants.

## Results

### The Effect of Quercetin on Sickling and P_sickle_ Activity

**Figure 1:**
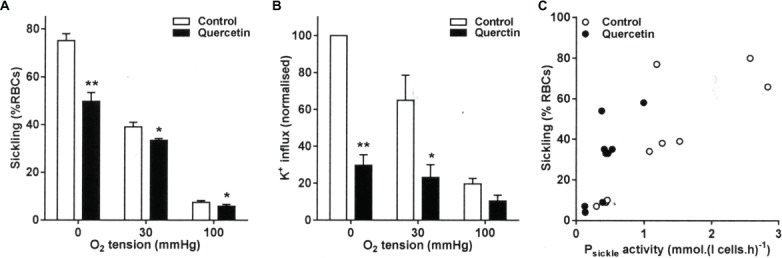
Effect of quercetin on sickling and P_sickle_ activity in red blood cells (RBCs) from sickle cell anemia (SCA) patients. RBCs were pre-incubated for 30 min at 37°C in air without or with quercetin (100 μM) in N-MBS. They were then equilibrated for 20 min in Eschweiler tonometers at the required O_2_ tension in the continued absence or presence of quercetin. Subsequently, **(A)** aliquots were fixed in glutaraldehyde (0.25%) and sickling determined by light microscopy. Histograms represent means ± SEM, *n* = 6. *p < 0.05 and **p < 0.01, comparing RBCs in the absence (control) and presence of quercetin. **(B)** Aliquots were diluted 10-fold into flux tubes (pH 7) and P_sickle_ activity measured for 10 min as K^+^ influx in N-MBS in the presence of clotrimazole (5 μM), bumetanide (10 μM), and ouabain (100 μM) at an extracellular [K^+^] of 7.5 mM and with influxes normalized to those of control RBCs at 0 mmHg. Histograms represent means ± SEM, *n* = 3. *p < 0.05, **p < 0.01, comparing RBCs in the absence and presence of quercetin. **(C)** Correlation between sickling and P_sickle_ activity. Pearson correlation coefficients are *r* = 0.83 (*p* < 0.01) and *r* = 0.77 (*p* < 0.02) in the absence and presence of quercetin, respectively. Symbols represent data from three individual patients at the three O_2_ tensions.

Quercetin is a flavonoid plant pigment. It is known to penetrate RBCs, where it binds to iron and also scavenges free hydroxyl and peroxyl radicals (Afanas’ev et al., 1989). Quercetin (100 μM) treatment significantly inhibited sickling at all three O_2_ tensions tested (full oxygenation, full deoxygenation, and at the intermediate O_2_ tension of 30 mmHg). At full deoxygenation, sickling was markedly reduced from 75 ± 3 to 50 ± 4% (Figure 1A, *p* < 0.01), while inhibition was still present though at smaller magnitudes at 30 and 100 mmHg O_2_. Quercetin treatment also markedly reduced P_sickle_ activity, although the effect was significant only at the intermediate O_2_ and in fully deoxygenated RBCs (Figure 1B, *p* < 0.05 and *p* < 0.01), and not at 100 mmHg O_2_. There was also a significant correlation between P_sickle_ activity and sickling in RBCs treated with quercetin (Figure 1C), consistent with the sickling shape change activating this conductance. P_sickle_ and sickling correlate strongly in untreated RBCs (*r* = 0.83, *p* < 0.01), and while quercetin reduces both sickling and P_sickle_ activity, this correlation remains (*r* = 0.77, *p* < 0.02).

### The Effect of Quercetin on Gardos Channel Activity

**Figure 2:**
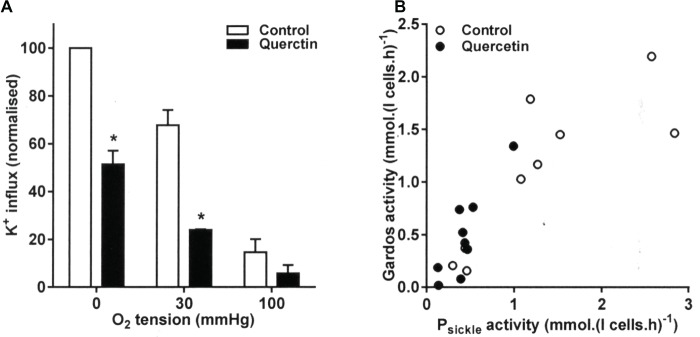
Effect of quercetin on Gardos channel activity in RBCs from SCA patients. RBCs were pre-incubated for 30 min at 37°C in air without or with quercetin (100 μM) in N-MBS. They were then equilibrated for 20 min in Eschweiler tonometers at the required O_2_ tension in the continued absence (control) or presence of quercetin. Gardos channel activity, defined as the clotrimazole (5 μM)-sensitive K^+^ influx in Cl-MBS, was measured in the continued absence or presence of quercetin. Ouabain (100 μM) and bumetanide (10 μM) were present, and influxes were normalized to those of control RBCs at 0 mmHg. **(A)** Histograms represent means ± SEM, *n* = 3. *p < 0.05, comparing RBCs in the absence and presence of quercetin. **(B)** Correlation between P_sickle_ (data taken from Figure 1) and Gardos channel activity. Pearson correlation coefficients are *r* = 0.82 (*p* < 0.01) and *r* = 0.88 (*p* < 0.005) in the absence and presence of quercetin, respectively. Symbols represent data from three individual patients at the three O_2_ tensions.

The effect of quercetin on Gardos channel activity showed the same relationship to that of P_sickle_. Quercetin decreased the level of Gardos channel activity (Figure 2A). The effect was most pronounced at the intermediate O_2_ tension and in fully deoxygenated RBCs, with activities declining from 1.21 ± 0.15 and 1.81 ± 0.25 mmol (l cells.h)^−1^ to 0.43 ± 0.05 and 0.94 ± 0.24 mmol (l cells.h)^−1^, respectively (both *p* < 0.05). These findings are consistent with an inhibitory of quercetin on sickling, indirectly reducing Gardos channel activity *via* the inhibition of Ca^2+^ entry through P_sickle_. Similar to sickling, Gardos channel activity correlated strongly to P_sickle_ activity (*r* = 0.82 and 0.88, without or with quercetin, *p* < 0.01 and *p* < 0.005, respectively, Figure 2B).

The Gardos channel can also be activated by pharmacological loading of RBCs with Ca^2+^ using the ionophore bromo-A23187. In this case, channel inhibition following quercetin treatment was still observed with *V*_max_ declining from 902 ± 133 to 482 ± 106 mmol (l cells.h)^−1^. Notwithstanding an indirect effect through a reduction in P_sickle_ activity, there was therefore also evidence for a more direct inhibitory action on the channel itself in Ca^2+^-loaded RBCs.

### The Effect of Quercetin on KCl Cotransport Activity

**Figure 3:**
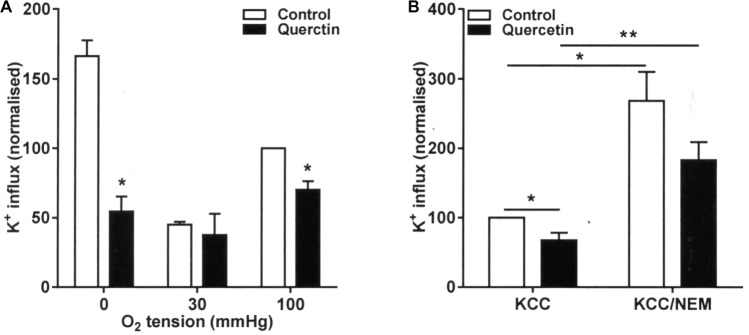
Effect of quercetin on KCl cotransport (KCC) activity in RBCs from SCA patients. RBCs were pre-incubated for 30 min at 37°C in air without or with quercetin (100 μM) in N-MBS. They were then equilibrated for 20 min in Eschweiler tonometers at the required O_2_ tension in the continued absence (control) or presence of quercetin. KCC activities, defined as the differences of clotrimazole-insensitive K^+^ influx in the absence and presence of Cl^−^, were measured. Ouabain (100 μM) and bumetanide (10 μM) were present in all experiments, and influxes were normalized to those of control RBCs at 100 mmHg. **(A)** RBCs were pre-incubated for 30 min at 37°C in air without or with quercetin (100 μM) in N-MBS, equilibrated in Eschweiler tonometers and KCC activity measured in the continued absence or presence of quercetin. Histograms represent means ± SEM, *n* = 3, *p < 0.05, comparing RBCs in the absence and presence of quercetin. **(B)** RBCs were first pre-incubated in N-MBS without or with NEM (1 mM) for 45 min at 37°C, 20% Hct. Both aliquots were divided and subsequently incubated without or with quercetin for 30 min, after which aliquots were diluted 10-fold into flux tubes and KCC activity measured as described in **(A)**. All incubations were carried out in air. Histograms represent means ± SEM, *n* = 5. **p < 0.01, comparing RBCs in the groups indicated.

The effect of quercetin was also examined on KCC activity at the three O_2_ tensions used to investigate its behavior on sickling, P_sickle_, and Gardos channel. In RBCs from patients with SCA, there is an aberrant response to O_2_ tension such that KCC activities are the highest and similar in fully oxygenated and fully deoxygenated RBCs while reaching at nadir at about the P_50_ for saturation of Hb with O_2_ (Gibson et al., 1998b). This is unlike the pattern in RBCs from normal individuals in which there is a monotonic decline in KCC activity with O_2_ tension (Gibson et al., 1998b). In the presence of quercetin, KCC activity was reduced at all O_2_, although the effect was not significant at the intermediate O_2_ tension (*p* = 0.7) when KCC activity was at its lowest (Figure 3A). The reduction was significant in both fully oxygenated and deoxygenated RBCs, falling from 1.68 ± 0.14 and 2.78 ± 0.12 to 1.19 ± 0.19 and 0.93 ± 0.27 mmol (l cells.h)^−1^, respectively (Figure 3A, *p* < 0.05 for both).

RBC KCC activity is thought to be controlled by conjugate pairs of regulatory protein kinases and phosphatases, such that it is stimulated by net dephosphorylation of the transporter itself or some regulatory protein. Activity of KCC increases following treatment with the thiol reacting reagent *N*-ethylmaleimide (NEM), an effect thought to be due to its action in reducing the effects of inhibitory protein kinases. This action of NEM was confirmed in the present work (Figure 3B, *p* < 0.01). Treatment with quercetin was able to inhibit KCC activity both before and after NEM treatment, although the latter did not quite reach significance (Figure 3B, *p* < 0.01 and *p* = 0.07). These results are consistent with inhibition by quercetin being mediated through effects on the regulatory phosphorylation step(s) and directly on the transporter *per se*.

### Effect of Quercetin on Hemolysis in Deoxygenated Non-electrolyte Solution

**Figure 4:**
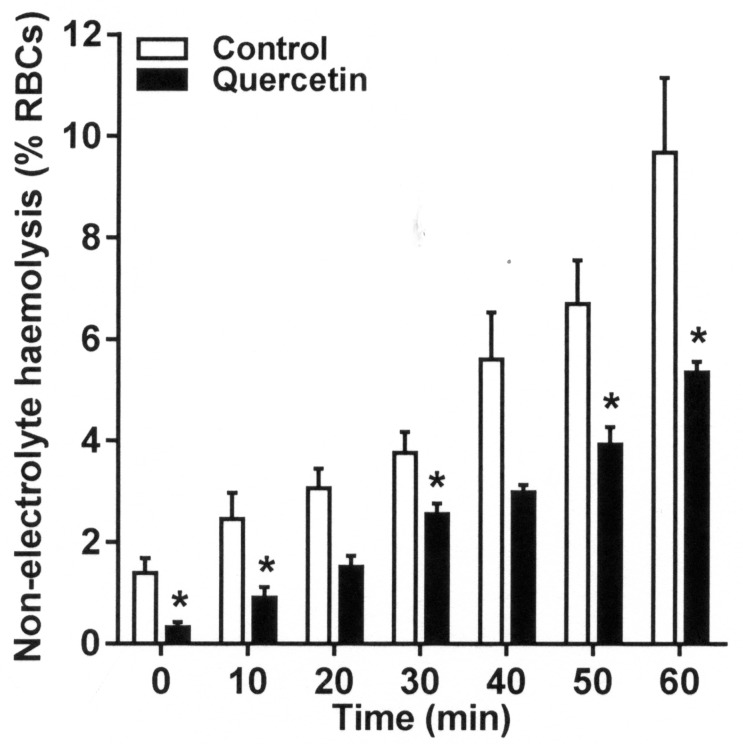
Effect of quercetin on hemolysis on RBCs from SCA patients in deoxygenated isosmotic sucrose solutions. RBCs were pre-incubated without or with quercetin in Ca^2+^-free N-MBS for 30 min. They were then incubated in isosmotic non-electrolyte solution in Eschweiler tonometers and equilibrated with N_2_ for 60 min. Hemolysis (% total RBCs) was measured at 10 min intervals by removing serial aliquots of RBC suspensions, pelleting intact RBC by centrifugation, and measuring the optical density (OD) of the supernatant at 540 nm. About 100% hemolysis was ascertained by measuring OD in RBC suspensions treated with X-100 Triton (0.1% final). Histograms represent means ± SEM, *n* = 4. *p < 0.05, comparing RBCs in the absence (control) and presence of quercetin.

RBCs undergo hemolysis when deoxygenated in isosmotic non-electrolyte solutions. Hemolysis often correlates with P_sickle_ activity and is taken as a measure of RBC fragility. The effect of quercetin on hemolysis was investigated in the present work. As expected from its effects on sickling and P_sickle_ activity, quercetin treatment reduced the rate of hemolysis in deoxygenated isosmotic sucrose solution. Inhibitory effects were apparent throughout the time course of the assay, consistently reaching about 40% after 20 min (Figure 4, *p* < 0.05), consistent with a stabilizing action on the integrity of the RBC membrane.

### The Effect of the Antioxidants Dithiothreitol and *N*-Acetylcysteine on Red Blood Cell Permeability

The effects of two other antioxidants dithiothreitol (DTT) and *N*-acetylcysteine (NAC) were also examined. DTT protects oxidized thiol groups and may reduce disulfide bonds. NAC is a cysteine pro-drug associated with protection of reduced glutathione (GSH) levels. In a similar way to quercetin, both significantly (p < 0.05) reduced sickling and the activities of P_sickle_, the Gardos channel, and KCC. As for quercetin, the latter two effects were also seen in RBCs treated with Ca^2+^ ionophore or pretreated with NEM. As these data are very similar to those obtained with quercetin, they are not repeated in full here, but effects are summarized in Table 1.

**Table 1:** A comparison of the effect of three antioxidants, dithiothreitol, *N*-acetylcysteine, and quercetin on the permeability of red blood cells (RBCs) from patients with sickle cell anemia.

Antioxidant	Sickling (0 mmHg)	P_sickle_ activity (0 mmHg)	Gardos channel activity (0 mmHg)	KCl cotransport activity (100 mmHg)	KCl cotransport activity (0 mmHg)	Hemolysis at 60 min
Dithiothreitol (*n* = 4–5)	−32 ± 8%	−58 ± 5%	−82 ± 8%	−31 ± 9%	−33 ± 6%	−69 ± 6%
*N*-acetylcysteine (*n* = 3–4)	−49 ± 6%	−51 ± 14%	−65 ± 13%	−43 ± 14%	−73 ± 14%	−50 ± 12%
Quercetin (*n* = 3–6)	−34 ± 6%	−70 ± 7%	−49 ± 7%	−30 ± 7%	−66 ± 10%	−42 ± 7%

*Sickling, P_sickle_ activity, and Gardos channel activity are given for RBCs incubated at 0 mmHg O_2_ while KCC activity is given at both 100 and 0 mmHg O_2_. The effect on hemolysis in isosmotic non-electrolyte solutions is given after 60 min deoxygenation. Values show the percentage inhibition on treatment with antioxidants. Comparing untreated RBCs and those treated with antioxidants, decreases in values were significant throughout (p < 0.05)*.

### The Effect of Antioxidants on Phosphatidylserine Exposure

**Figure 5:**
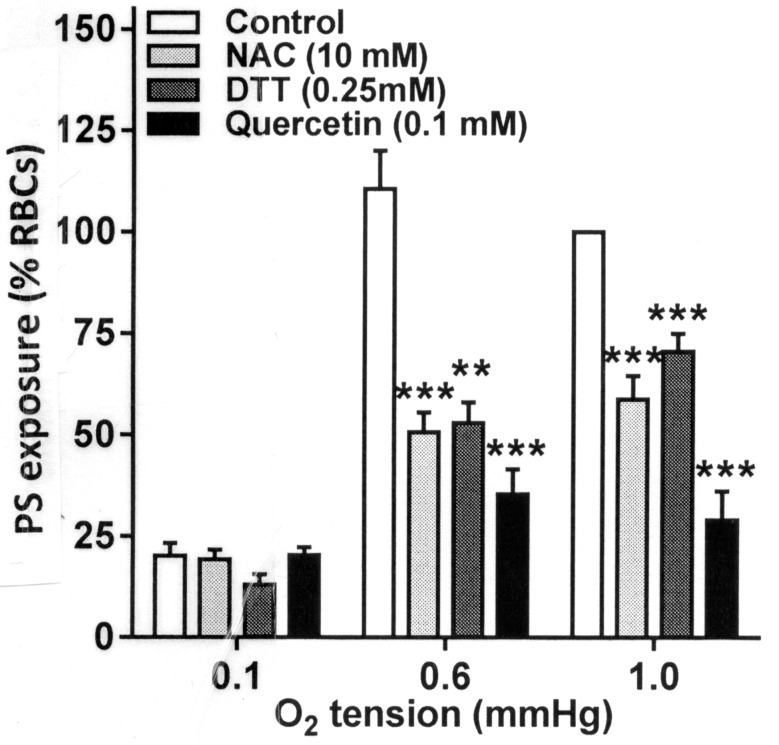
Effect of antioxidants on Ca^2+^-induced phosphatidylserine (PS) exposure in RBCs from SCA patients. RBCs were incubated in HK-HBS with bromo-A23187 and different free extracellular [Ca^2+^]s for 30 min in the absence (control) or presence of dithiothreitol (DTT, 0.25 mM, *n* = 10), *N*-acetylcysteine (NAC, 10 mM, *n* = 7) or quercetin (0.1 mM, *n* = 7). PS was then labeled with FITC-lactadherin and percentage of positive RBCs determined by flow cytometry. PS exposures were normalized to those of control RBCs incubated with a free extracellular [Ca^2+^] of 1 μM. The data for DTT have been previously published (Hannemann et al., 2018). Histograms represent means ± SEM for RBCs from *n* different patients. **p < 0.01, ***p < 0.001 compared to RBCs in the absence of antioxidant.

In the final series of experiments, the effect of antioxidants was tested on phosphatidylserine (PS) exposure. PS exposure was elicited by two maneuvers, Ca^2+^ loading using the ionophore bromo-A23187 and also by deoxygenation over 80 min. All three antioxidants, DTT, NAC, and quercetin, inhibited Ca^2+^-induced PS exposure (Figure 5, *p* < 0.01 and *p* < 0.001). Of the three, however, quercetin alone significantly reduced deoxygenation-induced PS exposure, completely abolishing it (*p* < 0.001).

## Discussion

The present findings show that three commonly used antioxidants, dithiothreitol (DTT), *N*-acetylcysteine (NAC), and quercetin, have significant beneficial effects on the membrane permeability of RBCs from patients with SCA. All three antioxidants reduced K^+^ permeability, inhibiting the three main transport systems associated with RBC dehydration – the KCl cotransporter (KCC), P_sickle_, and the Gardos channel. They also reduced phosphatidylserine (PS) exposure and hemolysis. In addition to their better appreciated role of protecting RBC proteins and lipids from oxidative damage, they therefore also reduced solute loss, RBC fragility, and PS exposure.

Although the etiology of SCA is simple – a mutated Hb with a single amino acid substitution – the disease has a complex pathogenesis affecting many body systems (Bunn and Forget, 1986; Steinberg, 1999; Rees et al., 2010). Pathologies are often grouped into two – a chronic hemolytic anemia with superimposed episodes of microvascular occlusion (Steinberg, 1999; Hebbel et al., 2004; Kato et al., 2009; Rees et al., 2010). Intravascular hemolysis is marked, with subsequent scavenging of nitric oxide (Gladwin et al., 2004).

Altered membrane permeability of HbS-containing RBCs is also an important feature and has been known about for over 60 years (Tosteson et al., 1952; Lew and Bookchin, 2005). Increased permeability to monovalent cation causes RBC shrinkage and associated elevation of HbS concentration, thus markedly encouraging polymerization (Eaton and Hofrichter, 1987; Lew and Bookchin, 2005). The high permeability to divalent cations, notably Ca^2+^ and Mg^2+^, has several effects (Ortiz et al., 1990; Etzion et al., 1993; Tiffert et al., 2003). Ca^2+^ entry activates the Gardos channel and further promotes solute loss (Etzion et al., 1993). Elevation of RBC intracellular Ca^2+^ concentration also stimulates PS exposure making RBC sticky and attractive to macrophages (Cytlak et al., 2011; Weiss et al., 2012). Exit of Mg^2+^ may serve to increase KCC activity (Delpire and Lauf, 1991). All of these encourage vascular adhesion and reduce RBC deformability, thus contributing to the pathogenesis of SCA.

A role of increased oxidative stress in pathogenesis is also anticipated (Rice-Evans et al., 1986). HbS is less stable than normal HbA and provides an intracellular oxidant challenge to RBCs, while extracellular oxidants come from activated white cells and the problems of ischemia/reperfusion (Hebbel et al., 1982, 1988; Rice-Evans et al., 1986; Banerjee and Kuypers, 2004). Oxidants damage the RBC membrane and cytoskeleton (Baskurt et al., 1998). They affect RBC deformability and rheology (Hebbel et al., 1990; Watanabe et al., 1990; Barodka et al., 2014). They have also been shown to stimulate solute loss and therefore increase RBC dehydration (Muzyamba et al., 2000; Gibson and Muzyamba, 2003a,b).

Antioxidants have therefore received considerable attention and their protective effects much studied. Three antioxidants in particular have been much studied – DTT, NAC (Gibson et al., 1998a; Shartava et al., 1999; Nur et al., 2012), and quercetin (Henneberg et al., 2013). DTT protects thiols and has previously been shown to reduce PS exposure (Hannemann et al., 2018). NAC maintains levels of reduced glutathione and reduces sickling and dense cell formation (Gibson et al., 1998a). It has been shown to reduce oxidative stress in sickle cell patients (Nur et al., 2012), although a more recent trial was inconclusive, perhaps because of non-compliance (Sins et al., 2016). Quercetin is able to sequester free radicals *via* ortho-dihydroxy structures (Moridani et al., 2003). It, too, protects against oxidative damage in sickle cells *in vitro* (Henneberg et al., 2013; Queiroz and Lima, 2013) and has been used clinically in cancer treatment (Ferry et al., 1996; M. D. Anderson Cancer Center, 2018).

The present results detail their effects on K^+^ permeability. Inhibition of the main dehydration pathways (P_sickle_, the Gardos channel, and KCC) will reduce solute loss, shrinkage, and hence the tendency for HbS to polymerize. They also inhibited Ca^2+^-induced PS exposure. The overall effect of all three antioxidants on RBC membrane permeability is therefore protective in several previously undocumented mechanisms.

An additional potential antioxidant is represented by L-glutamine. This reagent probably acts through increasing levels of reduced nicotinamide adenine dinucleotides and also possibly of reduced glutathione. It has received some attention for treatment of patients with SCA (Niihara et al., 2005, 2014a,b) and has been the subject of phase 3 clinical trials in which it appears to produce beneficial effects (Niihara et al., 2018). In our future work, we will address its effects on red cell permeability.

In conclusion, findings represent a further important rationale for provision of antioxidant therapy to patients with SCA.

## Data Availability

All datasets generated for this study are included in the manuscript and/or the supplementary files.

## Ethics Statement

All research was conducted with ethical approval (NHS REC 16/LO/1309) and in accordance with the Helsinki Declaration of 1975, as revised in 2008.

## Author Contributions

JG and DR designed the experiments. HA and AH carried out the experiments. JG, DR, JB, and AH contributed to writing the manuscript.

### Conflict of Interest Statement

The authors declare that the research was conducted in the absence of any commercial or financial relationships that could be construed as a potential conflict of interest.
